# BookTok: A Narrative Review of Current Literature and Directions for Future Research

**DOI:** 10.1111/lic3.70012

**Published:** 2024-11-22

**Authors:** Jeroen Dera

**Affiliations:** ^1^ Modern Languages and Cultures Radboud Universiteit Nijmegen Netherlands

**Keywords:** BookTok, digital media, literacy, narrative review, online reading platforms

## Abstract

Harvesting hundreds of billions of views, the hashtag #BookTok on TikTok is currently having a global impact on the production, distribution and reception of literature. This article assesses the academic research conducted thus far on this online literary phenomenon. It differentiates among three directions in the research: (1) BookTok as a specific form of book reviewing; (2) BookTok as a literary community‐builder; and (3) BookTok as an agent in reading promotion. Based on this narrative review, directions for future research are explored, with particular attention to the use of empirical methodologies to further investigate user experiences, the application of a celebrity studies lens, and a focus on BookTok accounts that combine affective and critical approaches to literature, exploring discussions on political, social, and environmental issues within the platform.

## 1 Introduction

Since early 2020, the phenomenon of #BookTok has become an integral part of the international literary field. This hashtag represents a subcommunity on the social media platform TikTok (established in 2017) where books are the central commodity. As of mid‐2024, over 35 million videos have been tagged with this hashtag globally, not to mention the millions of times the term is used on other social media platforms. Collectively, these posts have been viewed over 200 billion times. Despite the existence of literary communities on TikTok's predecessors Facebook and Instagram, BookTok has had an unparalleled impact on the production, distribution, and reception of literature, marking a unique chapter in the literary history of digital media.

For this reason, it is unsurprising that BookTok has garnered increasing academic attention in recent years. According to Google Scholar (as of September 15, 2024), there have been 1410 publications mentioning the phenomenon since 2020, compared to 1.080 for BookTube and 935 for Bookstagram. Given the expectation that this body of research will continue to expand in the coming years, and recognizing the significant interest BookTok holds for students in literary studies, literacy studies, and media studies, this article aims to narratively synthesize the emerging trends and insights from research on BookTok and to propose directions for future studies.

The review focuses on publications related to BookTok that have appeared in peer‐reviewed academic journals and conference proceedings since 2020. Specifically, it synthesizes and contextualizes the findings from 21 articles that explicitly address book‐related content on TikTok in their research questions or data sets (marked with an asterisk in the references section). These articles were selected by entering the term “BookTok” into the databases Google Scholar, Narcis, and Scopus, and include publications indexed in these databases as of summer 2024. The choice to focus on peer‐reviewed articles and conference proceedings ensures that the studies discussed meet the quality standards upheld by scholars in the field. This does not imply that research excluded from this review is of inferior quality; in fact, a wealth of student theses on BookTok, containing valuable insights, is available online. However, because it is difficult to assess the criteria used by various universities for making student theses publicly accessible (with marginal passes possibly being available alongside cum laude work), such publications have been excluded from this review.

In the following, I outline the current state of research on BookTok, focusing on three key areas that are frequently addressed in the articles under examination: (1) the nature and form of book reviews on BookTok; (2) the platform's community‐building function; and (3) BookTok's role in promoting reading in an era of declining readership. I connect these areas to broader insights from research on reading and online literary spaces in the 21st century. I finally propose potential avenues for future research, based on gaps in the existing literature and emerging trends on the platform at the time of writing.

## 2 Focus 1—The Nature and Form of Book Reviews on BookTok

The nature and form of BookTok videos are largely shaped by the technical specificities of TikTok as a social medium. TikTok, a platform owned by the Chinese conglomerate ByteDance, entered Western markets in mid‐2018, initially attracting a predominantly Generation Z audience by offering the ability to share very short, humorous videos and memes that integrate visuals, sound, and text. The platform quickly became central to the generational culture and identity of Gen Z, serving as a hub for various subcultures. This was facilitated by TikTok's algorithm, which groups users based on shared interests and behaviors. Alongside prominent communities like BookTok, TikTok hosts extensive subcultures focused on makeup, culinary arts, sports, and more. According to cultural studies scholars, TikTok differentiates itself from other social media platforms, particularly Facebook and Instagram, through its embrace of messiness, chaos, and playfulness, in contrast to the highly curated and filtered reality typically presented elsewhere (Jerasa and Boffone 2021). This openness to spontaneity fosters a sense of authenticity, especially appealing to adolescent users.

In line with TikTok's emphasis on multimodality, most BookTok videos contain a combination of spoken text, written text, music, and emojis, and viewers can engage with the videos by liking them, sharing them or leaving comments. As of mid‐2024, videos created using the TikTok app could be up to 60 s long, while those uploaded directly from a device could be up to 3 min. In an early content analysis of these short videos tagged with #BookTok, conducted in August 2020, Merga (2021) found that nearly half of the 116 videos in her corpus were so‐called recommendation videos, in which users provide specific book tips to their audience. This subgenre remained dominant in more recent research by Maddox and Gill (2023).

Characteristic of recommendation videos, also known in TikTok vernacular as “recs,” is that the creator aims to inspire their audience to read new books, often in the form of lists (e.g., “Books that are actually worth the hype,” “Best books I read this month,” “Best holiday picks”) or reading autobiographies (e.g., “Books that changed my life,” “All‐time favorite books”). A recommendation video can also be dedicated to a single book, although this overlaps with the BookTok review genre, where the video creator provides a more detailed discussion of the (usually recent) book and its merits. Such videos featuring specific reading experiences appeared in a quarter of the cases in Merga's (2021) corpus.

**FIGURE 1:**
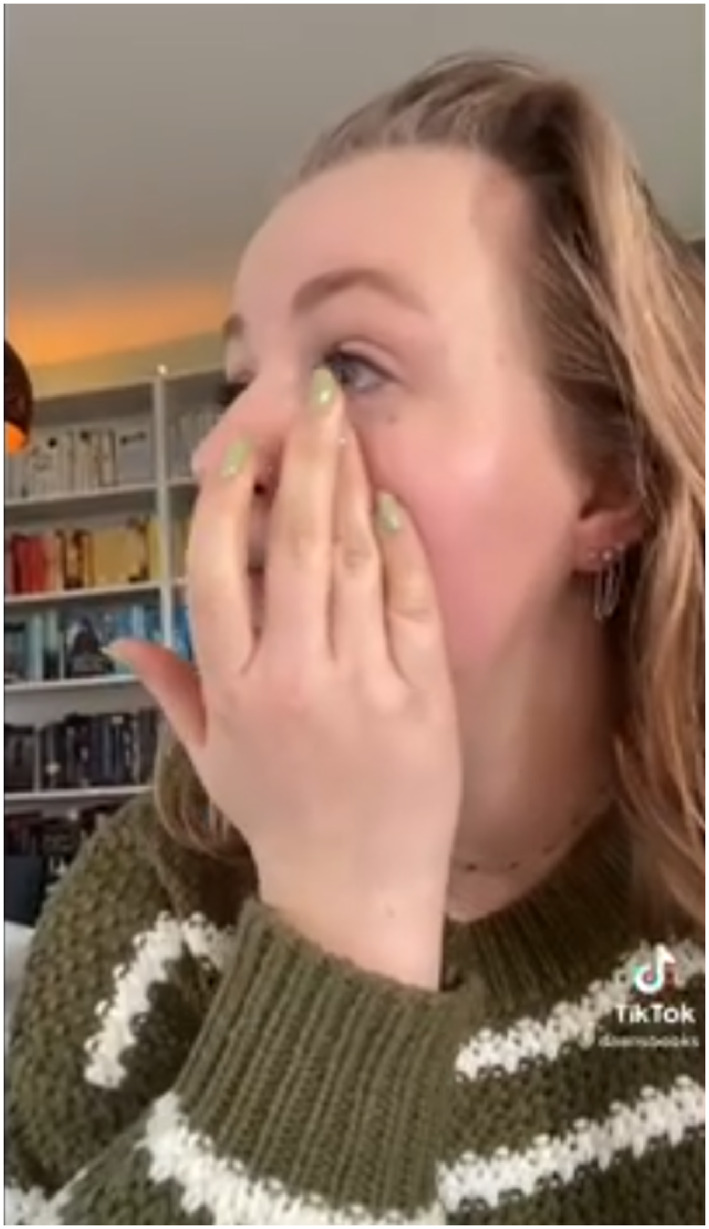
@daansbooks crying over *The Seven Husbands of Evelyn Hugo*.

Partly due to the emphasis on recommendations, which can sometimes take negative forms (e.g., “Books you won't get through”), book reviews on BookTok are overwhelmingly positive. On the platform, weighing between a positive or negative judgment is very rare. This is also related to the nature of literary‐critical evaluations on BookTok, which the Australian researcher Bronwyn Reddan (2022) describes as “visceral,” as if the judgments are deeply felt in the reader's body. Martens, Balling, and Higgason (2022) also describe this strong emphasis in BookTok reviews on emotions and affect, as opposed to a more rational distance regarding the thematic and stylistic properties of the books under consideration. Figure 1, for example, contains a still from a video of a BookTok creator tearfully recounting that she has finished reading *The Seven Husbands of Evelyn Hugo* (2017) by Taylor Jenkins Reid, sobbingly concluding: “Yes, good book.” As a reviewer, she radically foregrounds an authentic, affective reading experience, appealing to the relatability of this experience with her audience. This affective review style thus attains to identification within the community—an aspect of BookTok that is discussed in the next paragraph.

**FIGURE 2:**
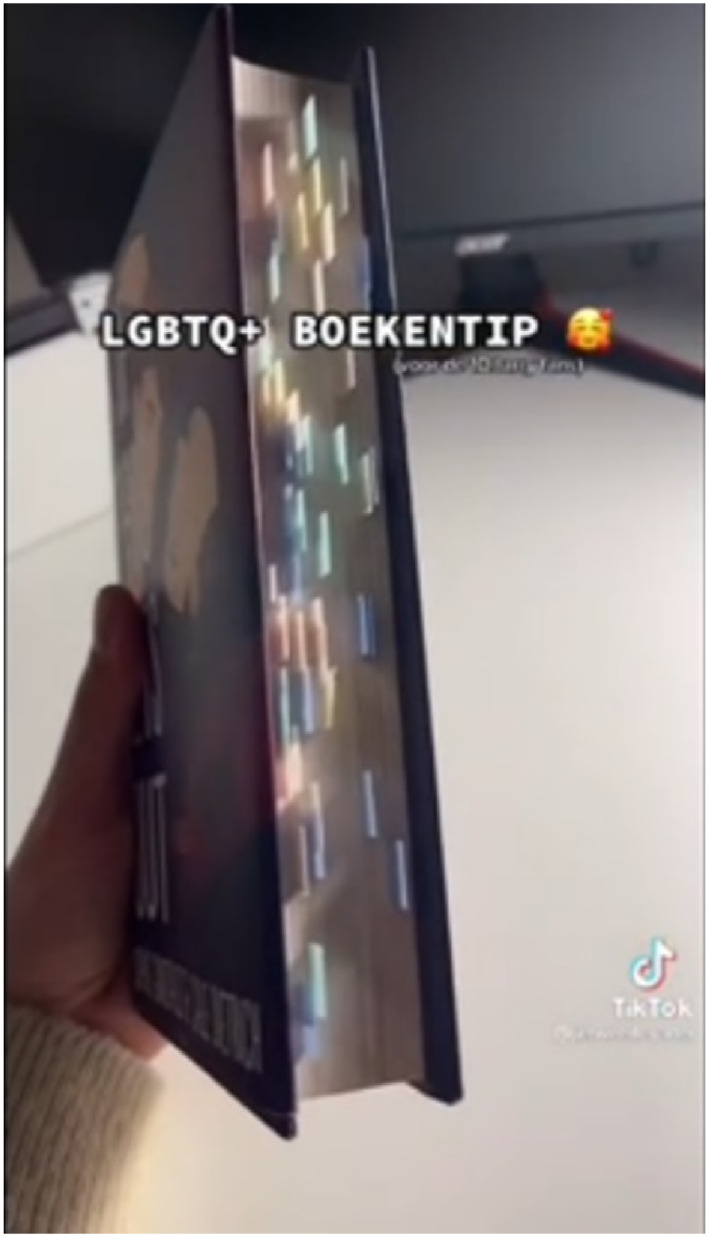
Example of “tabbing.”

The emphasis BookTok users place on emotion and affect is certainly not unique to the reading culture in digital media. Similar focuses have been described for Instagram, BookTube, and Goodreads (e.g., Pâquet 2019; Ehret, Boegel, and Manuel‐Nekouei 2018; Driscoll and Rehberg Sedo 2019), and the affective engagement with reading has strong historical roots (Birke 2023). A key aspect of this affective form of reading is the significant attention to the book as an artifact. As Martens, Balling, and Higgason (2022) extensively describe, reading on BookTok takes on a highly sensory and haptic form. Many videos depict the rustling of paper, mention the olfactory aspects of books through hashtags such as #booksmell, and highlight the physical comfort that reading can provide, for instance through images of special reading chairs or cushions. Additionally, many BookTok creators engage in literal bodily practices during reading, such as writing in books or “tabbing” them, where variously colored stickers are placed in the book at passages significant to the reader. In the videos, the focus is not so much on sharing the content of these passages but rather on showcasing the result of the tabbing process (see Figure 2). Recent interview research by Asplund, Egeland, and Olin‐Scheller (2024) indicates that this practice is inspiring to viewers of such videos, suggesting the influence of online representations of reading on actual reading behavior in the physical world.

An aspect of the tactile culture surrounding reading that has received special attention in research on BookTok is the phenomenon of the “shelfie,” a variant of the selfie in which users share images of their bookcases or specific shelves within them. This phenomenon is not unique to TikTok—the connotation of “selfie” and thus of photography already indicates the strong connection of shelfies with photo‐oriented media like Instagram—but it is widely applied in videos showcasing users' book collections. Dezuanni et al. (2022) point out the often highly stylized manner in which this occurs, with books arranged in particular ways (e.g., according to the colors of the rainbow) or surrounded by chic objects, thus not only aestheticizing but even glamorizing the books. Like the tabbing phenomenon, this luxurious engagement with physical books is a demonstrable source of inspiration for the young readers who participated in the empirical research by Asplund, Egeland, and Olin‐Scheller (2024).

This specific attention to the artifact has important implications for our understanding of book love as mediated through literary‐critical practices in BookTok videos. In this context, the aestheticization of the book as an object does not necessarily equate to its reading. Dezuanni et al. (2022) emphasize that while the videos of influential BookTok creators are highly composed and thus require a serious investment of time, they do not necessarily encourage the *reading* of books. Rather, they aestheticize book ownership and sometimes convey a discourse that highlights a different, yet still strongly affective aspect of the reading experience, framing reading as a struggle in times of overstimulation.

A relatively recent development in the research on the nature and form of reviewing on BookTok concerns the attention to TikTok's algorithm, specifically its role within videos and its impact on users. Low, Ehret, and Hagh (2023) conducted a multimodal content analysis of what they refer to as “algorithmic imagination” on BookTok. Drawing from the work of Taina Bucher, this concept refers to how people imagine, use, and experience algorithms, and what these imaginations enable in terms of critical distance from the dominance of the algorithm. In their research, Low, Ehret, and Hagh (2023) focused on videos that combined the hashtags #algorithm and #booktok and found that some community members explicitly questioned the TikTok algorithm in their videos (for instance, due to concerns about the intertwining of corporate interests and TikTok's platform logic) or humorously attempted to subvert it. Conversely, they also found many users who expressed that they were perfectly comfortable with the algorithm. Both groups, according to Low, Ehret, and Hagh (2023), contribute to the maintenance of the algorithm.

In the view of Jerasa and Burriss (2024), this algorithm is crucial for understanding the nature of BookTok videos. Reasoning from a critical posthumanist framework, which understands cultural forms on the internet as joint accomplishments between humans and machines and considers writing and texts as fundamentally nonneutral, they assert that BookTok consists of more‐than‐human assemblages. Therefore, analyses must always consider the role of artificial intelligence and the algorithm. Jerasa and Burriss (2024) demonstrate this through semi‐structured interviews with five BookTok users, interrogating their concrete interactions with the algorithm and showing that users can act *for*, *with*, and *against* it. In the first case, users make video choices that optimize their chances of getting many views based on the presumed workings of the algorithm (for instance, aligning with broader TikTok trends, which, according to Martens, Balling, and Higgason (2022), happens in many BookTok videos). In the second case, they use suggestions offered by the algorithm, such as matching music. Acting against the algorithm involves resisting its repressive effects, for example, by using words in contexts of books about homosexuality or drug use that the algorithm does not flag as harmful, with the goal of reaching the widest possible audience.

The study by Jerasa and Burriss (2024) illustrates how intertwined the literary‐critical BookTok practice is with the TikTok algorithm and how users perceive the latter as part of their “audience.” In this context, considering the broader cultural studies research on algorithms, the affective focus of BookTok reviews is particularly striking. In relation to Goodreads, Murray (2021) noted that the affective mode in literary criticism is strongly intertwined with capitalist algorithms, in the sense that the intimacy of positive emotions keeps people on the platform longer, thereby making them more attractive to advertisers. It is worthwhile to further investigate to what extent this observation also applies to BookTok. In her recent exploration of affective reader responses on BookTok, Kulkarni (2024) makes a first contribution to this perspective, convincingly demonstrating that the highly affective reviewing style on the platform is connected to the attention economy of the social medium and the imitative logic that arises from it.

## 3 Focus 2—BookTok as a Community

The TikTok algorithm connects and segregates users into categories or communities based on their (perceived) interests and behavior on the platform. According to Maddox and Gill (2023), this means that hyper‐specific objects (in this case books) serve as micro‐binds and further points of connection for individual users. The formation of a powerful subculture around books and reading on TikTok can thus be partly explained by the algorithm bringing together people who consider books an integral part of their lives, a phenomenon Birke (2021), building on Pressman's (2020) work, refers to as a “bookish identity.”

Current research reveals seven facets of this subcultural community, elaborated upon below:

1. **A young community **
  The BookTok community primarily consists of individuals, particularly women, from the millennial and Gen Z generations. Merga (2021) reports that most BookTok users in her 2020 sample did not disclose their age in their profile information, but among the approximately 40% who did, the age range was 13–27 years. In the past 2 years, the average age of TikTok users has increased slightly compared to 2020, which also appears to be reflected in the BookTok community, where women aged 25–34 dominate according to recent data from the United States and the United Kingdom (Townend 2024). While it is too early to draw definitive conclusions, this rising age demographic could indicate that the BookTok community is shifting away from adolescents, potentially altering the nature of the community over time.
2. **A community of readers without noise from non‐readers **
  Even though most BookTok users will never personally meet other community members, there is a sense of community due to the shared interests on which TikTok groups its members and the sense of relatability that accompanies this (Maddox and Gill 2023). In this respect, BookTok parallels online affinity spaces, as defined by Curwood (2013): the platform offers users a choice in their level and means of participation, the ability to enjoy multiple modes of representation, and an authentic audience responding to their work. Authenticity is particularly crucial for young users, as they often seek book recommendations from relatable peers (Loh and Sun 2022). For adolescent readers, BookTok provides a space free from the negative noise of classmates who dislike reading and express this disdain indiscriminately (Merga 2021).
3. **A community with a canon of its own **
  In the early years of BookTok, there was a strong emphasis on young adult titles in users' repertoires, corresponding with the average age of its community (Merga 2021). In recent years, the proportion of young adult literature has decreased in favor of genres such as (dark) romance, fantasy, and spicy novels—which might be related to the growing average age of users on the platform. While literary titles are indeed represented, they are rarer in the selection. Thus, to the extent that a BookTok canon exists, it diverges from the traditional literary canon emphasized in education (Jerasa and Boffone 2021). Maddox and Gill (2023) note that new members must familiarize themselves with the works of certain authors to stay informed about BookTok trends, creating a hierarchy within the offerings *and* the community. For example, authors such as Colleen Hoover, Sarah J. Maas, and Adam Silvera might be essential for staying current. In other words, a minimum repertoire is required for full participation. Simultaneously, such a canon facilitates a strong sense of community and serves as a starting point for the algorithm to recommend titles that align with popular trends, a feature technically exploited through pre‐filled texts in search bars (e.g., “they both die at the end recommendations”). According to Maddox and Gill (2023, 7), this “strengthens the bond of an online community through suggesting similar content.”
4. **A non‐monolithic community **
  Although much is written about “the” BookTok community, it is definitely not a monolithic entity. Instead, BookTok comprises numerous subcommunities, such as #SapphicBookTok and #AsianBookTok. As these niches become more specific, their content is more likely to diverge from broader literary‐critical trends on BookTok (as described in the previous paragraph). For instance, Dera (2024a) demonstrated that poetry accounts and analyses of the hashtag #sylviaplath on the platform combine an affective approach to poems with a more rational and ideological engagement with the genre.
5. **A community giving voice to underprivileged groups **
  Popular subcommunities on BookTok include #BlackBookTok, #QueerBookTok, and #JewishBookTok, among others. The popularity and high visibility of queer‐themed titles within the broader community's repertoire (e.g., Alice Oseman's *Heartstopper* series, Aiden Thomas' *Cemetery Boys*, Alison Cochrun's *The Charm Offensive* series) particularly contribute to LGBTQIA+ members feeling more represented on this platform compared to other book‐based social media. Hence, Boffone and Jerasa (2021) argue that BookTok facilitates online community formation for underprivileged groups, such as the queer community. However, Maddox and Gill (2023) identify a notable tension between many users' lived experiences, where BookTok is perceived as highly inclusive regarding the representation of marginalized groups, and the algorithm's reality, which predominantly shows new users content targeted at white, cisgender, heterosexual women, including a focus on titles with limited diversity.
6. **A transnational community with an Anglophone bias **
  BookTok is an international phenomenon, and while the hashtag is also translated to other languages (e.g., the Spanish #librotok, the Dutch #boektok, the German #buchtok), the English hashtag is widely adopted across borders. Most cited research on BookTok originates from English‐speaking regions, too, although Martens, Balling, and Higgason (2022) are Danish, Dera, Brouwer, and Welling (2023) are Dutch, Asplund, Egeland, and Olin‐Scheller (2024) are Swedish, and Guiñez‐Cabrera and Mansilla‐Obando (2022) are Chilean. Asplund, Egeland, and Olin‐Scheller (2024) note that Swedish youth primarily engage with English‐language books on BookTok and exhibit a strong preference for reading in English. A similar trend is observed in other Western European countries, either anecdotally or empirically (e.g., Weber 2024, on Germany; Dera and Van Doeselaar 2022, on the Netherlands; Snaije 2024, on France). This shift is driven by BookTok and other online literary phenomena, such as Goodreads reading challenges (compare Dera 2024b). BookTok's transnational aspect, highlighting the strong cultural dominance of English, warrants further attention in future research, which should also pay attention to the BookTok phenomenon in non‐western contexts.
7. **A community transcending virtual boundaries **
  Finally, while BookTok is quintessentially an online affinity space, current research highlights its offline community‐building extensions as well. Participants in Asplund, Egeland, and Olin‐Scheller (2024) indicated that connecting with others was a major motivation for their BookTok use, empirically confirming what Maddox and Gill (2023) proposed theoretically. The teenagers interviewed by Asplund, Egeland, and Olin‐Scheller (2024) reported that they genuinely *felt* this community and experienced it beyond TikTok—such as at school, where they discussed BookTok titles with friends or classmates. This demonstrates that the community‐building facilitated by BookTok transcends virtual boundaries.

In conclusion, current research indicates that the BookTok community exhibits a dual nature. On one hand, it displays homogeneity through a shared repertoire, gender dynamics, the conceptualization of books as a lifestyle, and a strong sense of belonging to a group with common ideas about the significance of books in their lives. On the other hand, it is highly diverse, encompassing specific subcultures, genres, and spanning across various countries.

## 4 Focus 3—BookTok and Reading Promotion

BookTok, particularly in its early years, is predominantly a bottom‐up phenomenon that emerged organically among young readers, rather than being imposed by adults with literacy‐promoting intentions. Its immense popularity has now attracted significant focus from the book market and educational sector, drawing increasing attention from researchers.

According to Nicoll (2023), the “BookTok effect” refers to the platform's ability to boost a book's popularity and sales through TikTok. This effect is evident not only in the sustained rise in sales of viral titles but also in the creation of dedicated BookTok sections in bookstores and libraries. From a literacy promotion perspective, it is intriguing (yet economically understandable) that publishers and booksellers target youth who already enjoy reading or did so as children. BookTok catalyzes further growth in their reading motivation or helps them rediscover the joy of reading during adolescence, a period when they often lose interest and suffer declines in intrinsic reading motivation (cf. Asplund, Egeland, and Olin‐Scheller 2024; Dera, Brouwer, and Welling 2023).

Such campaigns within the BookTok community present new opportunities for publishers. Chiovelli and Cameron (2023) and Segarra‐Saavreda and Torres‐Huamanyauri (2023) illustrate how publishers leverage BookTok for targeted marketing, communicating with existing and potential audiences through their own accounts, and sending titles to influential BookTokers for review. Nicoll (2023) adds that BookTok can generate buzz around new titles, particularly through advance reader copies (ARCs) used by BookTok influencers to enhance their status within the community. Additionally, an increasing number of authors are active on TikTok, though it remains under‐researched how they use the platform in practice. Yet, by directly networking with their target audience and integrating into the community, both publishers and authors can ensure sustained engagement with their publications, creating what Ackermans (2021) terms “branded readers.” It must be emphasized, though, that this top‐down intervention risks disrupting the community's bottom‐up structure (Wiederhold 2022).

Concerning libraries, the BookTok effect raises specific challenges. While it allows librarians to present themselves in the community, enabling them to better interact with users, as shown by Mashiyane (2022) for academic libraries, public libraries face two challenges in particular: managing a large volume of similar‐genre books for a balanced collection, and handling the rapid turnover of popular titles. Chiovelli and Cameron (2023) discuss strategies for either acquiring many copies of trending books or adopting alternative approaches.

In the context of literature education, BookTok presents intriguing questions as well. Jerasa and Boffone (2021), early advocates for integrating BookTok into literacy classrooms, highlight its potential for fostering student agency, reading choices, and student voice in digital space unregulated by teachers. They advise using BookTok videos for classroom analysis, possibly combined with literary‐analytical tools and discussions on representation, for instance focusing on marginalized voices that acquire agency in BookTok subcommunities. They also encourage teachers to create their own content and design reflective exercises for students in which they are asked to do the same. Such advocacy appears to resonate with school librarians, for recent research by Moore, Evans, and Schultz‐Jones (2023) indicates that they, too, are tracking developments on BookTok and particularly encourage older students to share reviews in online environments.

The literature largely expresses optimism about BookTok's potential for reading promotion as well. Jerasa and Boffone (2021) suggest that BookTok might attract new readers, Dezuanni (2021) views it as a catalyst for developing a reader identity, and Merga (2021) cautiously anticipates it could improve the negative perception of reading among youth. Empirical insights are provided by Dera, Brouwer, and Welling (2023), who studied 173 ninth‐grade students in the Netherlands exposed to seven BookTok videos that were highly popular during data collection. The analysis revealed that especially avid readers were drawn to BookTok, while those with a negative reading attitude were significantly not. At the same time, so‐called book doubters—students with a neutral to positive reading attitude who nevertheless do not read frequently—showed relatively positive evaluations compared to book avoiders. Still, this latter group appeared enthusiastic about the incorporation of BookTok videos in literature classes, suggesting that attention to BookTok could positively impact their motivation and appreciation for literacy lessons.

Despite these results, a persistent tension remains between the titles young BookTok users enjoy for pleasure and their use in educational contexts, where literature is often required to be complex and resistant to decoding (e.g., Nikolajeva 2010; Dera 2024c). While popular genre fiction on BookTok can encourage reading amidst declining behavior, reconciling this with the educational goal of developing readers proficient with complex texts is still an open question. A specific concern in this regard is whether outspokenly literary texts might be further marginalized by the algorithm, raising the barrier to such texts even in educational settings. This relates to English, Enderle, and Dhahecka's (2023) warning for “binge‐reading,” where readers repeatedly consume the same type of texts over and over again, because the algorithm continues to recommend them. In this context, literacy promoters, such as educators and (school) librarians, should also spotlight complex literary titles to the BookTok audience. In doing so, they can leverage the popularity of current trends, for instance through pointing *Heartstopper* fans to the poster of Evelyn Waugh's classic Brideshead Revisited that protagonist Charlie has on his bedroom door (Dera 2024d).

## 5 Directions for Future Research

While the previous sections primarily reviewed recent research, I will now outline potential directions for future research on BookTok. The synthesis above already highlighted several aspects that require further exploration through rigorous study: the transnational nature of the BookTok community, the behavior of literary and popular fiction authors active on the platform, and the complex tension between popular titles on BookTok and the development of literary reading skills in educational contexts. However, I will now delve more deeply into three additional areas that I believe are essential to any research agenda on BookTok. These areas arise partly from gaps in the literature discussed and partly from (emerging) trends on the platform that have yet to receive academic attention. Naturally, I do not claim that these suggestions encompass all possible directions for further research, but I hope they will inspire (new) researchers in the academic community with an interest in BookTok.

Firstly, it would be worthwhile to conduct more empirical research on BookTok users. As this review reveals, studies utilizing elicitation data generated among actual BookTok users are relatively rare, and when conducted, they often involve small sample sizes: eight teenagers in the case of Asplund, Egeland, and Olin‐Scheller (2024); five in the case of Jerasa and Burriss (2024). While such limited samples allow for an in‐depth description of the interviewed participants' experiences, they are still considered small for interview research, given that theoretical saturation in this type of research typically occurs with sample sizes between 9 and 17 participants, as shown in the recent study by Hennink and Kaiser (2022).

Therefore, larger samples are recommended for future interview research on BookTok users. Additionally, other empirical methodologies could be employed to gain more insight into how BookTok is used in practice. Mobile ethnography, in particular, is promising. This method involves participants sharing what they read in print and on their devices (smartphones, e‐readers, laptops, tablets) over a specific period through an app (Loh 2023). These data are often combined with in‐depth interviews, where concrete reading practices (including photo elicitations based on the app) from the studied period are presented to participants to gain insights into their behaviors, routines, and choices.

Questionnaire designs have so far only been used by Dera, Brouwer, and Welling (2023) and Moore, Evans, and Schultz‐Jones (2023), but these studies focus on high school students, most of whom did not use BookTok, and on school librarians, respectively. A large‐scale survey among BookTok community members might provide more insight into usage motives, user experiences, and sense of belonging on the platform. It is also suitable for examining whether significant differences exist between various groups of users or subcommunities active on BookTok. An especially interesting group for more rigorous investigation, including in interview research or a multiple case study design, is boys who are active on the platform. Questions include why cisgender, heterosexual boys are generally less attracted to the medium and whether opportunities exist to change this—also considering the recent popularity of BookTok videos where men who conform to beauty standards promote spicy romance fiction. Another population warranting further study is older BookTok users (Generation X, baby boomers). How do their usage motives, experiences, and practices compare to those of younger generations, to whom current BookTok research is exclusively directed? An additional reason to closely examine this population is the observed increase in the average age of users on the platform.

Secondly, it seems relevant to consider BookTok from the perspective of celebrity studies, a field focusing on the study of celebrities, both in their representation in various media and in the (discursive and institutional) ways media help shape fame (Elliott 2018). In the literature reviewed for this contribution, popular content creators are already referred to as micro‐celebrities (e.g., Dezuanni et al. 2022; Maddox and Gill 2023), but a systematic study of what such fame actually means within the BookTok community, the general book world, or the possibilities for reading promotion is currently lacking, to mention just a few potential areas for future research.

A celebrity studies lens could also provide insights into how bookfluencers emerge and operate, and shed light on how popular authors promote their work via BookTok and build a large fan base, such as spicy romance author Ana Huang or TikTok poet Celia Martinez. Additionally, research into celebrities outside the literary world who connect their stardom with promoting books on BookTok and other social media, such as film star Reese Witherspoon or top tennis player Iga Swiatek, remains unexplored. Such research on specific case studies would align well with the qualitative methodologies employed in many of the studies reviewed here and requires a rigorous immersion of the researcher into both the BookTok subculture and the fandom surrounding the celebrity in question.

The third and final direction for future research demands qualitative inquiry as well and concerns the extensive study of BookTok accounts and literary hashtags where the affective focus typical of BookTok is less present or combined with a more cognitive, (ideologically) critical approach to books and literary representation. Given the massive presence of recommendation videos on the platform, it is understandable that research has so far mainly focused on how this subgenre takes shape. However, various discussions about the literary canon, the book industry, and the political reality are conducted on BookTok that deserve further academic analysis. This includes, amongst many others, BookTok videos or comments addressing racism or its representations in books, BookTok videos focusing on ecocritical literature or climate fiction, or videos using the hashtag #womeninfiction to shape contemporary feminism in relation to literature.

The three perspectives mentioned above can equally be extrapolated to the study of other social media where users share and shape both their reading experiences and the way literature functions in contemporary literary media culture. Given the political controversies in the United States surrounding TikTok and its Chinese parent company ByteDance, it remains to be seen how the medium will develop in the short term. However, that it has left an undeniable mark on how particularly younger generations engage with literature through BookTok is beyond question—and as I have shown, there remains a significant scope for discovery in future research.
